# Effects of high‐fat diet‐induced adipokines and cytokines on colorectal cancer development

**DOI:** 10.1002/2211-5463.12751

**Published:** 2019-11-18

**Authors:** Jian Zhang, Shikui Guo, Jinyuan Li, Weimin Bao, Peng Zhang, Yingguang Huang, Ping Ling, Yongzhi Wang, Quan Zhao

**Affiliations:** ^1^ Department of General Surgery I The First People’s Hospital of Yunnan Province The Affiliated Hospital of Kunming University of Science and Technology China; ^2^ Medical Faculty Kunming University of Science and Technology China

**Keywords:** adipokine, colorectal cancer, cytokine, high‐fat diet, *in vitro*, *in vivo*

## Abstract

Colorectal cancer (CRC) is the third most common tumor worldwide, and recent epidemiological studies have indicated that obesity contributes to the morbidity and mortality of CRC. Furthermore, obesity‐related adipokines have been shown to be closely related to the incidence of CRC, but the underlying mechanisms are unclear. Here, we investigated the effects of high‐fat diet‐induced adipokines and cytokines on the development of CRC *in vitro* and *in vivo*. For the *in vivo* assays, we divided 2‐week‐old C57BL/6J‐ApcMin/J male mice into three groups: normal‐fat diet (ND), high‐fat and high‐sugar feed (HFHS), and high‐fat and low‐sugar feed (HFLS). After 1 week, all mice were injected with 20 mg·kg^−1^ 1,2‐dimethylhydrazine once weekly for 10 consecutive weeks. Body weight, liver weight, epididymal fat weight and blood glucose levels were greatly increased in HFHS and HFLS groups compared with the ND group, and the expression levels of some adipokines and cytokines were obviously higher in HFHS or HFLS mice compared with ND mice. For the *in vitro* assays, HCT116 CRC cells were treated with sera of ND, HFHS or HFLS groups, or serum‐free media as a negative control. We observed that sera derived from HFHS or HFLS mice that contain excess adipokines and cytokines promoted the proliferation, migration and invasion of HCT116 cells compared with the ND sera‐conditioned medium or serum‐free medium group. Therefore, high‐fat diet‐induced adipokines and cytokines may promote the progression of CRC *in vivo* and *in vitro*.

## Abstract 2

We investigated the effects of high‐fat diet‐induced adipokines and cytokines in the development of colorectal cancer. Some adipokines and cytokines were obviously higher in obese mice than in normal mice. Obesity‐induced adipokines and cytokines promoted the proliferation, migration and invasion of HCT116 cells. Therefore, high‐fat diet‐induced adipokines and cytokines may accelerate the progression of colorectal cancer.

## Graphical abstract

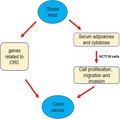

**Abbreviations**
CCK‐8: Cell Counting Kit‐8
CRC: colorectal cancer
CXCL‐10: chemokine‐10
DMEM: Dulbecco’s modified Eagle medium
DMH: 1,2‐dimethylhydrazine
HFHS: high‐fat and high‐sugar feed
HFHS‐CM: HFHS sera‐conditioned medium
HFLS: high‐fat and low‐sugar feed
HFLS‐CM: HFLS sera‐conditioned medium
IL‐6: interleukin‐6
MMP‐9: matrix metalloproteinase‐9
ND: normal‐fat diet
ND‐CM: ND sera‐conditioned medium
NF‐κB: nuclear factor‐κB
qRT‐PCR: quantitative RT‐PCR
SDF‐1: stromal cell‐derived factor‐1
SFM: serum‐free medium
TNF‐α: tumor necrosis factor‐α
VEGF: vascular endothelial growth factor

Colorectal cancer (CRC) is the third most common tumor worldwide. The number of new cancer cases has been increasing in the previous 10 years; consequently, the mortality rate has been growing 1. Recent epidemiological studies confirmed that obesity is one of the reasons responsible for the increasing morbidity and mortality associated with CRC 2, 3. The survey results of the US Health Professionals Follow‐Up Study suggest that overweight is a modifiable cause of CRC in men 4. According to Norwegian health reports, obesity during childhood and adolescence results in a 2‐fold increase in colon cancer‐related mortality in both men and women 5.

Adipose tissues secrete numerous active substances termed adipokines, including adiponectin, leptin, resistin, interleukin‐6 (IL‐6), among others. 6. Adipokines physiologically regulate development, metabolism, eating behavior, fat storage, insulin sensitivity, hemostasis, blood pressure, immunity and inflammation 7. Pathologically, excessive accumulation and secretion of adipokines in adipose tissue of obese patients have also been associated with other diseases, such as rheumatoid arthritis and osteoarthritis 8, intervertebral disc degeneration 9 and cardiovascular disease 10. Notably, proinflammatory cytokines, which are stimulated by adipokines 11, 12, bridge obesity and the aforementioned diseases. Research has shown that obesity‐related adipokines were closely related to the incidence of CRC 13.

Compared with normal colonic mucosa, the expression of leptin in colorectal adenoma or adenocarcinoma is significantly increased, indicating that the expression of leptin may be involved in the development and progression of CRC 14. Furthermore, it has been recently shown that adiponectin may be a prognostic parameter in determining the risk for CRC relapse 15. Recently, a study reported that CXCR4 is closely related to colon cancer. Stromal cell‐derived factor‐1 (SDF‐1) from the CXC chemokine family is a chemoattractant cytokine widely involved in physiological regulatory processes, including cell signaling. CXCR4 is activated by SDF‐1 to increase vascular permeability, producing a variety of tumor cell lines that adhere to extracellular matrix substrates and vascular endothelial cells 16. It is well established that adiponectin is one of the adipocytokines and is homologous to tumor necrosis factor‐α (TNF‐α). TNF‐α is closely associated with the occurrence and development of cancer, including cellular transformation, promotion, survival, proliferation, invasion, angiogenesis and metastasis. Importantly, its circulating levels may determine the disease status of patients with CRC 17.

However, previous studies have focused on the analysis of clinical patient data, with limited evidence for experimental analysis. Therefore, the purpose of this study was to further confirm the connection of high‐fat diet‐induced, obesity‐related adipokines or cytokines with the development of CRC.

## Materials and methods

### Animals and chemicals

In this study, 60 C57BL/6J‐ApcMin/J male mice (2 weeks old) were obtained from the Model Animal Research Center of Nanjing University (Nanjing, China) and used in the animal experiments. All mice were maintained in cages under the fixed conditions of humidity (44 ± 5%), light (12‐h light/dark cycle) and temperature (22 ± 2 °C). 1,2‐Dimethylhydrazine (DMH) was purchased from Sigma‐Aldrich (St. Louis, MO, USA) and prepared with 1 mm ethylenediaminetetraacetic acid saline (pH 7.0).

### Animal experimental procedures

All C57BL/6J‐ApcMin/J male mice (2 weeks old) were divided into three groups: a normal‐fat diet (ND) group, a high‐fat and high‐sugar feed (HFHS) group, and a high‐fat and low‐sugar feed (HFLS) group. Before the experiment, the mice were fed a normal diet and water for 1 week to adapt to their new environment. After acclimation, the mice were fed either an ND, HFHS [45 kJ% fat (soybean oil: 225 kJ; lard: 1598 kJ), 35% carbohydrate, 20% protein, D12451; Xietong, Nanjing, China; high‐sucrose content (30%) in the drinking water] or HFLS [45 kJ% fat, 35% carbohydrate, 20% protein, D12451 (Xietong); low‐sucrose content (10%) in the drinking water]. According to the different groups, fresh diets were provided diurnally to allow the mice to feed freely. At 3 weeks of age, all mice were intraperitoneally injected with DMH at a dose of 20 mg·kg^−1^ once weekly for 10 weeks in series. At the end of week 12, the mice were euthanized 18. The body weights of the mice were measured weekly and recorded throughout the experiment. The experimental protocol was approved by the Animal Care and Use Committee and the Ethics Committee of the First People’s Hospital of Yunnan Province (Kunming, China).

### Cell culture and preparation of culture medium

HCT116 CRC cells were obtained from the Cell Bank of Type Culture Collection of Chinese Academy of Sciences (Shanghai, China) and maintained in Dulbecco’s modified Eagle medium (DMEM; Invitrogen, Carlsbad, CA, USA) with 10% FBS (Sigma Chemical Co.) in a humidified incubator at 37 °C with an atmosphere of 5% carbon dioxide.

For *in vitro* experiments, cells were treated with four different sera: (a) serum‐free medium (SFM), (b) ND sera‐conditioned medium (ND‐CM), (c) HFHS sera‐conditioned medium (HFHS‐CM) and (d) HFLS sera‐conditioned medium (HFLS‐CM). The ND‐CM, HFHS‐CM or HFLS‐CM consisted of SFM plus 2.5% mixed sera from the corresponding group of mice.

### Necropsy and collection of samples

All mice were anesthetized through intraperitoneal injection of ketamine at a dose of 100 mg·kg^−1^ plus xylazine 15 mg·kg^−1^. The heart of each mouse was punctured to collect blood samples, and the serum was separated and stored at –80 °C until analysis 19. The levels of serum glucose in the three groups were estimated with a glucose oxidase method using a Hitachi autoanalyzer based on the instructions provided by the manufacturer 20. The entire colon was removed, washed with cold saline and collected for subsequent use. The tumors were removed from the colons and immediately frozen in liquid nitrogen for use in the real‐time quantitative RT‐PCR (qRT‐PCR) and western blotting experiments. In addition, the liver and epididymal fat were collected and weighed.

### ELISA

The levels of serum adiponectin, leptin, visfatin, IL‐6, TNF‐α and chemokine‐10 (CXCL‐10) were measured using corresponding ELISA kits (RD, Minneapolis, MN, USA) according to the instructions provided by the manufacturer.

### Real‐time qRT‐PCR

Total RNA was extracted from colons using the TRIzol (Invitrogen) method. After purification of RNA using the RNeasy kit (74104; Qiagen, Hilden, Germany), its concentration was determined, and 1 μg RNA from every sample was reverse transcribed into cDNA using the High Capacity cDNA kit (4368814; Applied Biosystems, Foster city, CA, USA). Real‐time PCR was performed using the Power SYBR green PCR master mix (Applied Biosystems) and ABI 7500 PCR machine (Applied Biosystems) (cDNA served as a template). Each sample was tested in triplicate. The mean cycle threshold from the qRT‐PCR was used to calculate the fold change; glyceraldehyde‐3‐phosphate dehydrogenase was used as a reference gene.

### Western blotting

Protein was extracted from colonic tissues, and its concentration was quantified using the BCA Protein Assay Kit (Pierce Biotechnology, Rockford, IL, USA). Subsequently, 10% SDS/PAGE was used to isolate the proteins, which were transferred onto polyvinylidene difluoride membranes (Millipore, Bedford, MA, USA). The polyvinylidene difluoride membranes were blocked for 2 h with 5% skim milk in Tris‐buffered saline at room temperature. The membranes were incubated under the condition of 4 °C for 8 h with specific antibodies against corresponding proteins [SDF‐1, ab9797; CXCR4, ab181020; vascular endothelial growth factor (VEGF), ab32152; matrix metalloproteinase‐9 (MMP‐9), ab38898; Abcam, Cambridge, MA, USA]. Next, the membranes were washed twice with Tris‐buffered saline containing 0.1% Tween 20 (10 min each wash). Subsequently, the appropriate horseradish peroxidase‐conjugated secondary antibody (1 : 5000 dilution) was used to incubate the membranes at room temperature for 1 h, and the membranes were washed according to the previous method. Finally, the proteins were detected through the enhanced chemiluminescence method (ECL kit; Pierce) based on the instructions, and NADP was used as an internal control.

### Cell Counting Kit‐8 proliferation assay

Cell Counting Kit‐8 (CCK‐8; Beyotime, Beijing, China) was used to detect the proliferative ability of cells. HCT116 cells were collected, and a cell suspension was seeded in 96‐well plates with SFM (5 × 10^3^ cells/well); three replicate wells for each sample were included. After incubation for 18 h, the culture medium was gently removed, and cells were treated with SFM, ND‐CM, HFHS‐CM or HFLS‐CM, respectively, at 37 °C. Cells were collected after treatment for 24, 48, 72, 96 or 120 h with different group sera. CCK‐8 reagent (10 µL) was added to each well, and the cells were incubated for 1 h at 37 °C. Finally, the optical density was determined at 450 nm using a microplate luminometer reader.

### Colony formation assay

HCT116 cells in the growth stage were digested with 0.25% trypsin; they were repeatedly blown to make the cells fully dispersed, and a cell suspension was prepared. Subsequently, the cells in the suspension were counted and seeded in a six‐well plate at a density of 1 × 10^3^ cells/well with complete medium. We prepared a new medium comprising DMEM supplemented with 2.5% FBS and either SFM, ND‐CM, HFHS‐CM or HFLS‐CM in advance. The following day, the medium was gently removed, and the new medium was added to the six‐well plate. The cells were incubated for 2 weeks following previous protocols 21 at 37 °C with an atmosphere of 5% carbon dioxide. During this time, the old medium was changed according to the change in its color. At the end of the treatment, the medium was gently removed from each of the wells, and the cells were washed twice with PBS at working concentration (1× PBS). Subsequently, the cells were fixed with 1% formaldehyde for 15 min and dried. Cells were stained with crystal violet, and the excess crystal violet was washed away with double‐distilled water. The plates were allowed to dry, and qualitative digital images of the colonies were captured, scanned and analyzed.

### Wound healing assay

We used a marker pen to draw a horizontal line on the back of the six‐well plate with a ruler. HCT116 cells were plated in six‐well plates (5 × 10^4^ cells/well) with culture medium. After 24 h, the medium was replaced with SFM. Horizontal lines were drawn with a sterile pipette vertically, and the detached cells were washed twice with PBS. Finally, SFM, ND‐CM, HFHS‐CM or HFLS‐CM was added into the six‐well plate, and the cells were incubated for 48 h. After treatment, images were captured at 0 and 48 h using a Nikon TE 2000 inverted microscope (magnification ×40) connected to a Nikon Coolpix 4500 camera (Nikon Corporation, Tokyo, Japan).

### Matrigel–Transwell invasion assay

Before the experiment, Matrigel mixtures were thawed at 4 °C overnight. The Matrigel mixtures were diluted with serum‐free DMEM and added to the upper chamber of a 24‐well Transwell (100 µL/well). The Transwell was incubated for 4 h at 37 °C. HCT116 cells were collected and suspended in media containing 1% FBS at a density of 1.2 × 10^5^ cells/mL, and cell suspension (200 µL) was added onto Matrigel. The lower chamber of the Transwell was filled with culture media containing SFM, ND‐CM, HFHS‐CM or HFLS‐CM as a chemoattractant. After 48 h, the noninvasive cells from the upper chamber were removed using cotton swabs. Subsequently, cells were stained with 1.0% crystal violet for 10 min at room temperature, and the images were captured using a Nikon TE 2000 inverted microscope connected to a Nikon Coolpix 4500 camera (Nikon Corporation). Acetic acid (33%) was used to counterstain cells, and the optical density was determined at 570 nm using a microplate reader.

### Statistical analysis

The results are shown as the mean ± standard deviation. All mice were divided into different groups in a blinded manner, as described in a previous study 22. The difference between groups was analyzed with one‐way ANOVA followed by a least significant difference *post hoc* test. A *P*‐value <0.05 denoted statistical significance.

## Results

### HFHS or HFLS feeding increases total body weight, liver weight, blood glucose levels and epididymal fat weight

**Figure 1:**
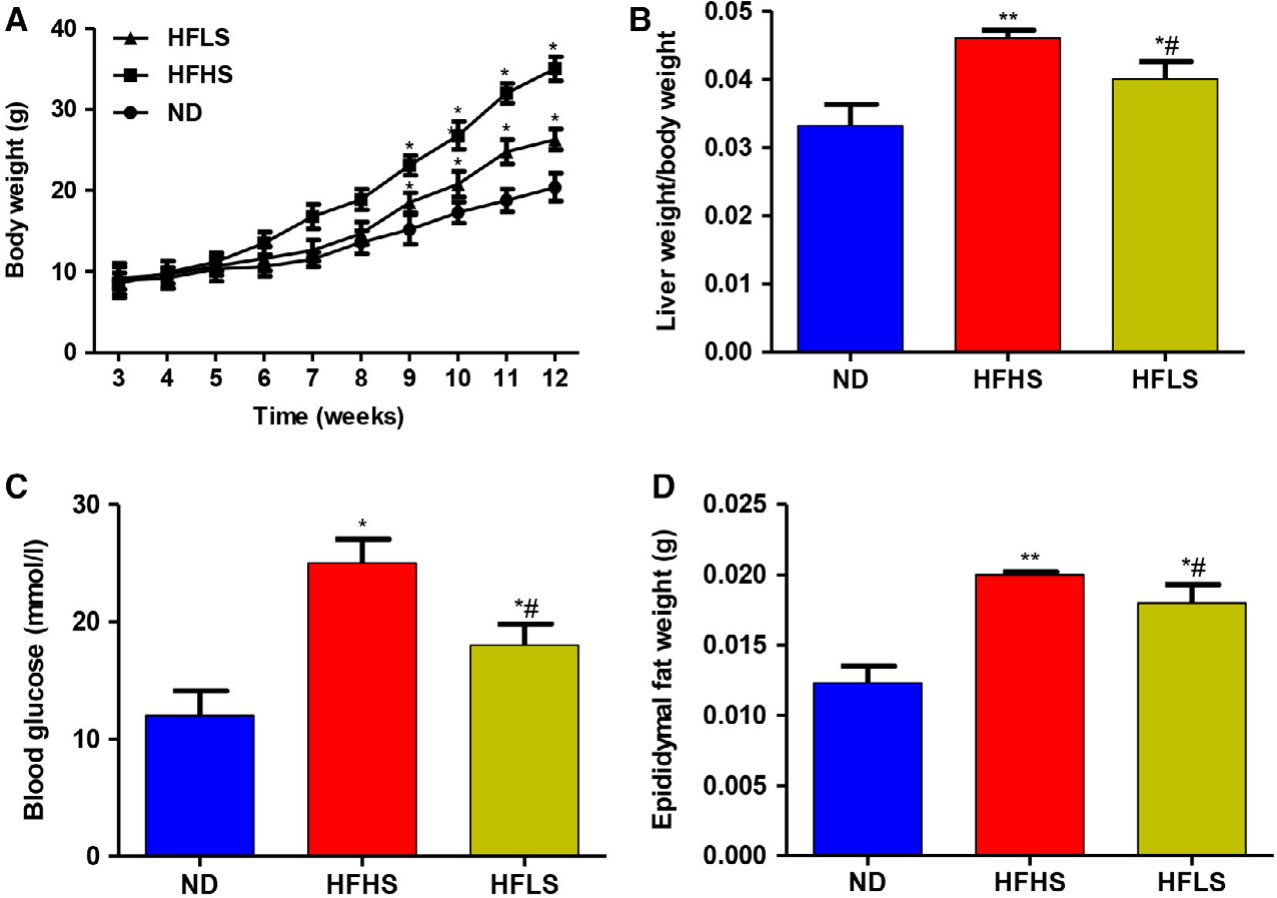
Changes in body weight, liver weight, blood glucose levels and epididymal fat weight. (A) Changes in body weight of mice fed an ND, HFHS diet and HFLS diet. (B) Average weight of the liver in the three groups. Each column represents the mean ± standard deviation. (C) Average value of blood glucose levels in the three groups. (D) Average weight of epididymal fat in the three groups. Each column represents the mean ± standard deviation (one‐way ANOVA; **P < 0.01, *P < 0.05, ND versus HFHS or HFLS; ^#^ *P* < 0.05, HFHS versus HFLS).

Glycometabolism and lipid metabolism are closely associated 23, 24, 25. Therefore, to provide more information through our study, we used HFHS and HFLS in this study. All mice survived to the end of the study (12 weeks). Figure 1A shows that the body weight obviously increased in the HFHS and HFLS groups at 9 weeks of age compared with that reported in the ND group. There was a difference between the HFHS or HFLS group and the ND group from 9 to 12 weeks of age. In addition, compared with the ND group, there was also a significant increase in liver weight (Fig. 1B), blood glucose levels (Fig. 1C) and epididymal fat weight (Fig. 1D) in the HFHS and HFLS groups. In addition, the liver weight, blood glucose level and epididymal fat weight in the HFLS group were markedly lower than those measured in the HFHS group.

### HFHS or HFLS feeding alters the levels of inflammatory cytokines and adipocytokines in the serum

**Figure 2:**
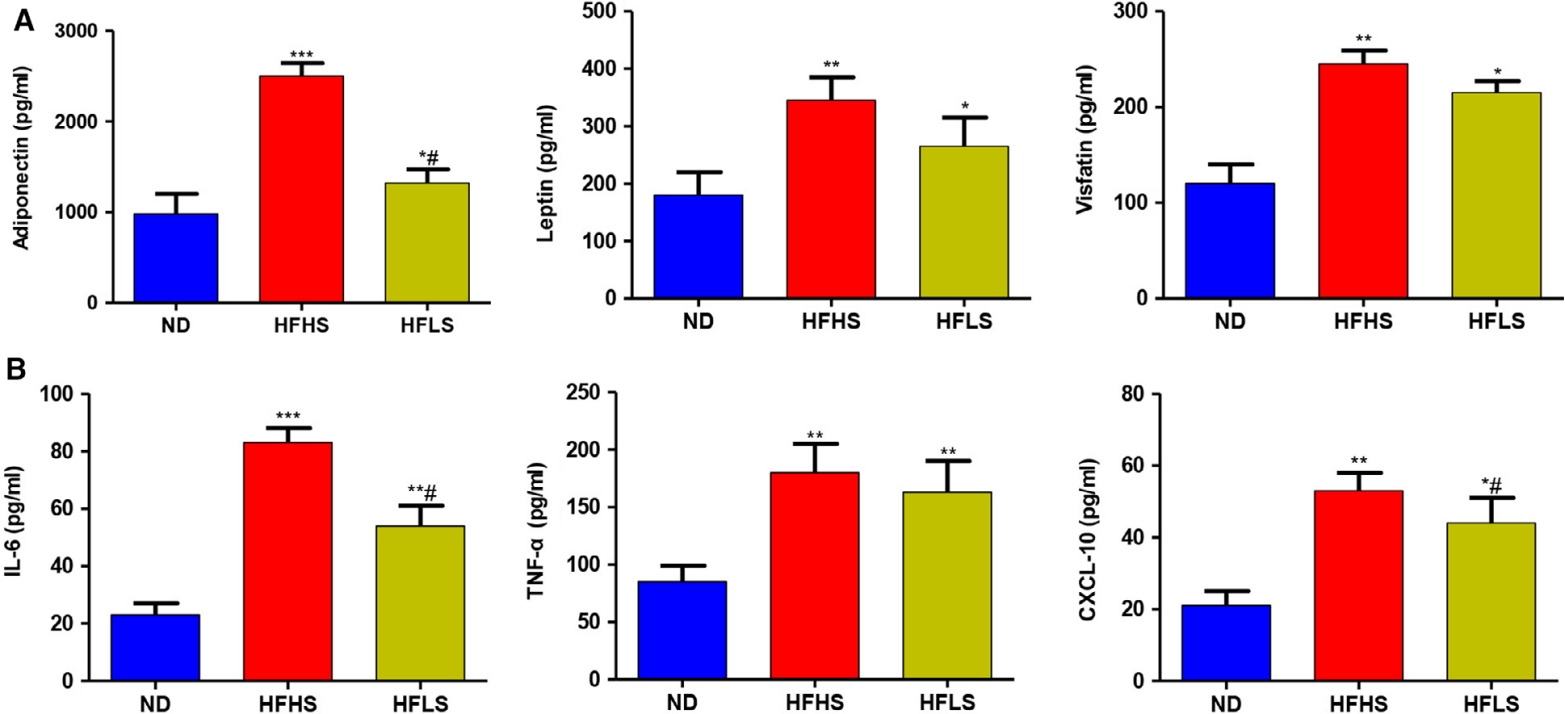
Levels of inflammatory cytokines and adipocytokines in the serum in the three groups. Each column represents the mean ± standard deviation. (A) Levels of adiponectin, leptin and visfatin in the serum. (B) Levels of IL‐6, TNF‐α and CXCL‐10 in the serum (one‐way ANOVA; ***P < 0.001, **P < 0.01, *P < 0.05, ND versus HFHS or HFLS; ^#^ *P* < 0.05, HFHS versus HFLS).

Adipose tissue secreted numerous inflammatory cytokines, such as adiponectin, leptin and visfatin. We found that the concentrations of adiponectin, leptin and visfatin in the serum increased in the HFHS‐fed and HFLS‐fed groups compared with the ND‐fed group (Fig. 2A). Furthermore, there was a significant increase in the levels of TNF‐α, IL‐6 and CXCL‐10 in the HFHS and HFLS groups compared with the ND group (*P* < 0.01; Fig. 2B).

### HFHS or HFLS feeding enhances the expression of genes related to colon cancer

**Figure 3:**
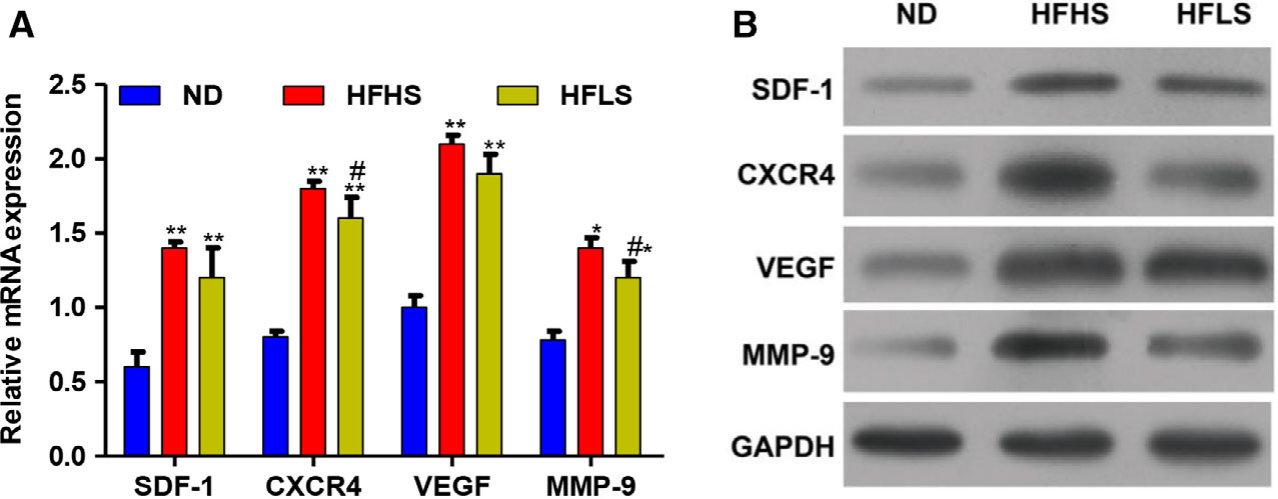
C57BL/6J‐ApcMin/J mice fed HFHS or HFLS demonstrate enhanced expression of genes related to colon cancer. (A, B) The expression of SDF‐1, CRCX 4, VEGF and MMP‐9 at the mRNA level (A) and the protein level (B) in the three groups (one‐way ANOVA; **P < 0.01, *P < 0.05, ND versus HFHS or HFLS; ^#^ *P* < 0.05, HFHS versus HFLS).

The expression of genes related to CRC (i.e., *SDF‐1*, *CRCX 4*, *VEGF* and *MMP‐9*) at the mRNA and protein levels was detected in the tumor tissues of mice. As shown in Fig. 3A,B, the expression levels of *SDF‐1*, *CRCX 4*, *VEGF* and *MMP‐9* in HFHS‐fed or HFLS‐fed mice were obviously higher than those measured in ND‐fed mice.

### Serum from HFHS‐fed or HFLS‐fed mice promotes proliferation and colony formation in HCT116 cells

**Figure 4:**
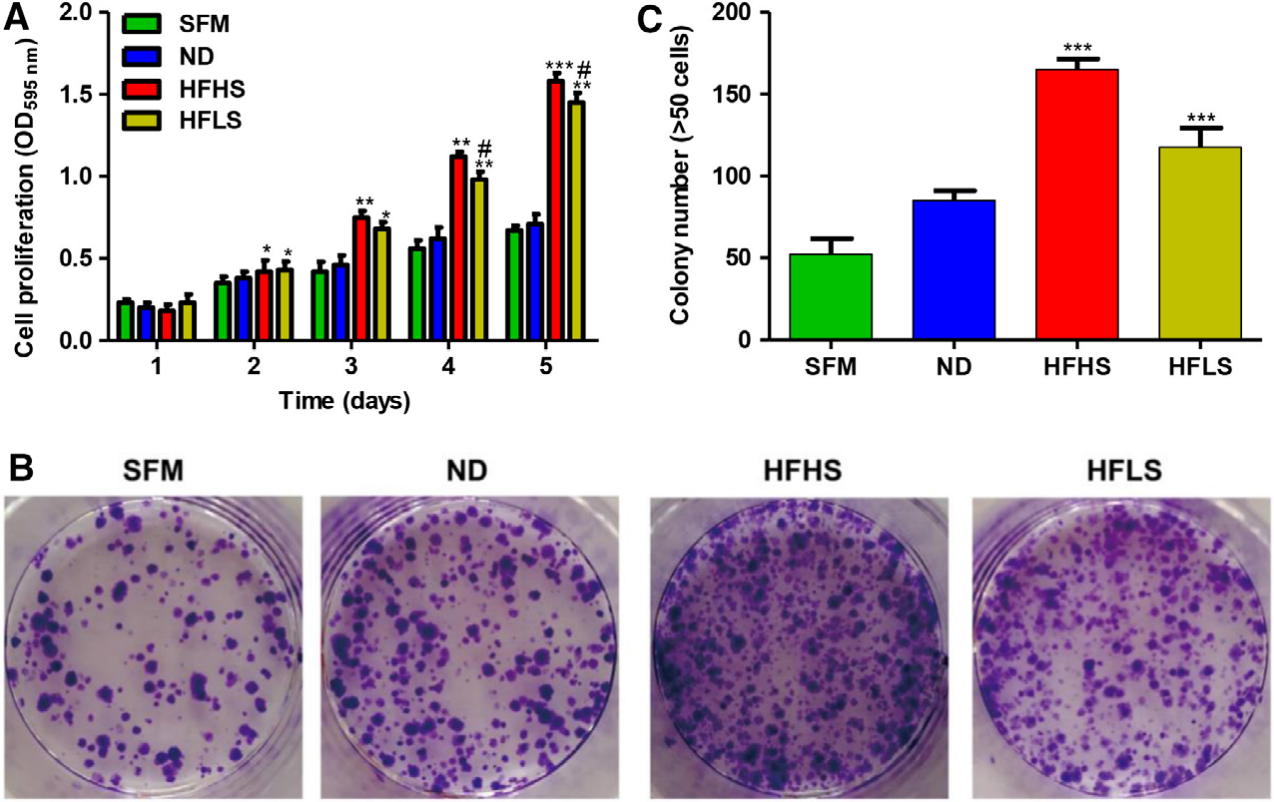
Serum from HFHS‐fed or HFLS‐fed mice promotes proliferation and colony formation. (A) HCT116 cells were treated with SFM or conditioned medium containing 2.5% mixed sera from ND‐fed, HFHS‐fed or HFLS‐fed mice. Cell viability was determined using the CCK‐8 assay. One‐way ANOVA was conducted. (B, C) In the colony formation assay, HCT116 cells were seeded 1 × 10^3^ cells/well, and data were presented as colony numbers. Results are presented as the mean ± standard error of the mean (*n* = 3; one‐way ANOVA; *P < 0.05, **P < 0.01, ***P < 0.001, ND versus HFHS or HFLS; ^#^ *P* < 0.05, HFHS versus*.* HFLS).

The proliferative ability of HCT116 cells after different dietary interventions was detected using the CCK‐8 proliferation assay. The results showed that, after 2 days of culture, the proliferation of HCT116 cells was faster in the HFHS‐CM and HFLS‐CM groups than in the SFM and ND‐CM groups (Fig. 4A). In the colony formation assay, cells treated with HFHS‐CM or HFLS‐CM formed more colonies compared with the SFM and ND‐CM groups (Fig. 4B,C), showing the acceleration effect of HFHS‐CM or HFLS‐CM on the clonogenic ability of colon cancer cells.

### Serum from HFHS‐fed or HFLS‐fed mice promotes the migration and invasion of HCT116 cells

**Figure 5:**
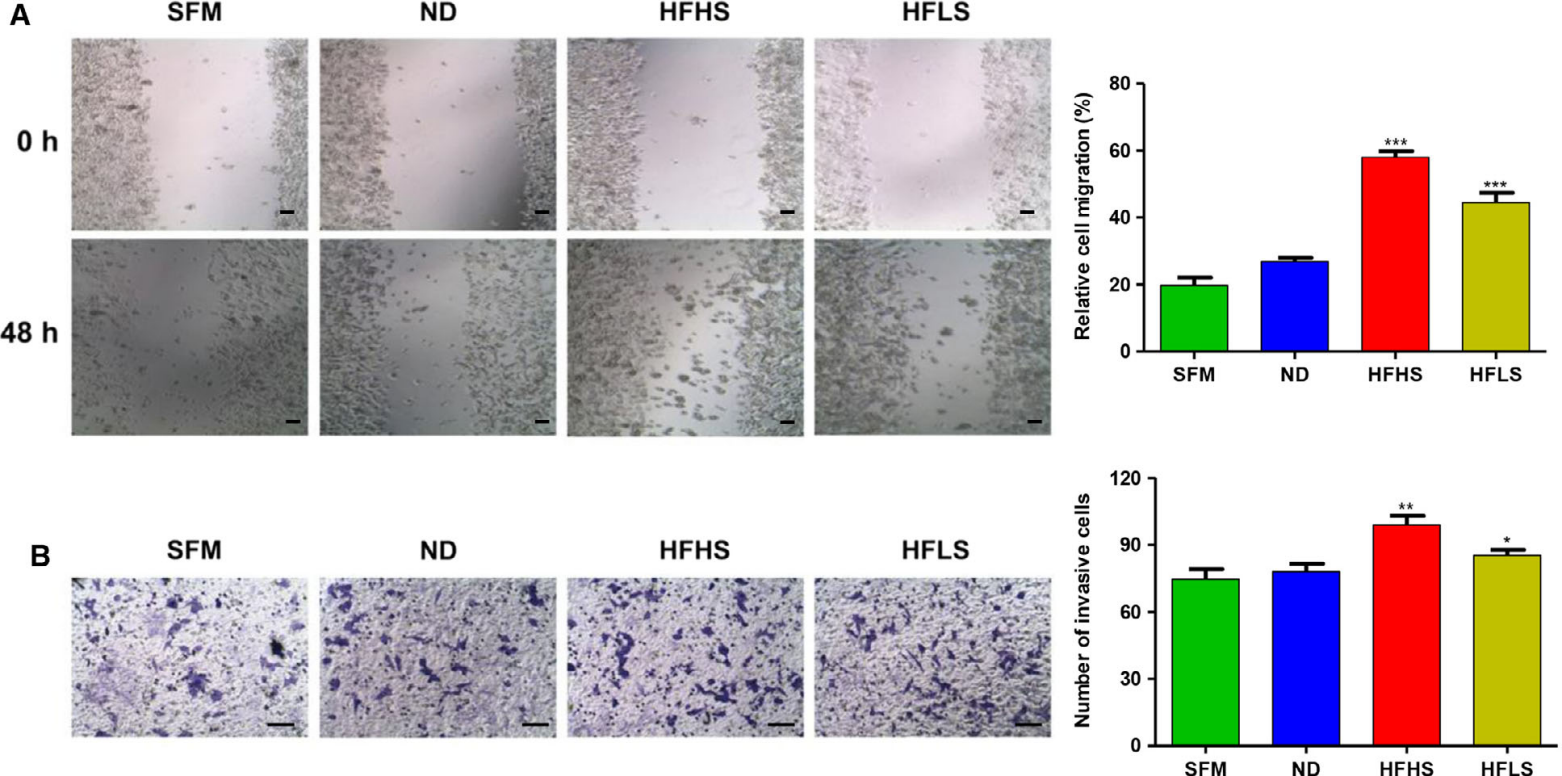
Serum from HFHS‐fed or HFLS‐fed mice promotes the migration and invasion of HCT116 cells. (A) Effects of SFM, ND‐CM, HFHS‐CM and HFLS‐CM culture on the migration of HCT116 cells were determined using a wound healing assay and representative images of the wound healing experiment. (B) Invasion of HCT116 cells was determined using a Matrigel–Transwell invasion assay. SFM, ND‐CM, HFHS‐CM or HFLS‐CM was added to the lower chamber of the Transwell as the chemoattractant, and representative images from the experiment are shown (one‐way ANOVA; *P < 0.05, **P < 0.01, ***P < 0.001, ND versus HFHS or HFLS). Scale bars: 200 μm (A); 100 μm (B).

After 48 h, cells cultured in HFHS‐CM or HFLS‐CM showed an increase in the speed of migration compared with those cultured in ND‐CM or SFM (Fig. 5A). In the Matrigel–Transwell assay, after culture for 48 h, there was a greater number of invasive cells in the HFHS‐CM and HFLS‐CM groups versus the ND‐CM and SFM groups (Fig. 5B).

## Discussion

In recent years, various diseases caused by obesity have attracted increasing attention of the national and international public health authorities. Particularly, obesity is closely related to some chronic illnesses, such as coronary heart disease, diabetes, and the occurrence and progression of tumors 26. It is generally accepted that obesity is related to the development and prognosis of CRC. The development of obesity is characterized by excess delivery of nutrients to the adipose tissues and an expansion in adipose mass, which disrupts the dynamic role of adipocytes in energy homeostasis. This process alters adipose‐derived hormones and leads to the development of chronic inflammation 27. Thus, in this study, we established a DMH model using C57BL/6J‐ApcMin/J male mice fed with an HFHS or HFLS diet to observe the body fat index and obtain serum samples to examine the expression levels of obesity‐induced adipokines and cytokines. The results showed that the average body weight, liver weight and epididymal fat weight were markedly increased in mice fed an HFHS or HFLS diet compared with those measured in mice fed an ND. These results are consistent with those reported by Zhu *et al*. 28. In that study, sustained intake of a high‐calorie or high‐fat diet significantly thickened the epididymal fat pads in mice, leading to obesity. The process of obesity in mice is similar to that observed in humans 29.

White fat stores excess energy for use when necessary. White adipose is also an endocrine organ that produces many adipokines (e.g., adiponectin, leptin and resistin), which are involved in diabetes and cardiovascular disease 30. Cytokines, mediated by the transcription factor nuclear factor‐κB (NF‐κB), are associated with tumors. NF‐κB is activated by multiple stimuli, including secreting hormones (e.g., leptin and adiponectin), inflammatory molecules (e.g., IL‐6, TNF‐α and IL‐1B) and growth factors (e.g., insulin‐like growth factor‐1 and VEGF), which are of particular relevance to the pathogenesis of CRC 31. Visfatin was originally identified as a pre‐B cell colony‐enhancing factor by Samal *et al.* 32 Pre‐B cell colony‐enhancing factor, secreted by leukocytes in the human body, is considered to be another form of visfatin. It upregulates the expression of other cytokines (e.g., TNF‐α, IL‐1B and IL‐6), also promoting the differentiation of B cells 26. In this study, we found that the concentrations of adipokines and cytokines in the serum were significantly higher in the HFHS and HFLS groups than those recorded in the ND group. Huang *et al.* 35 reported that the expression level of SDF‐1 is positively regulated by visfatin. In our study, the expression levels (mRNA and protein) of SDF‐1, CXCR4, VEGF and MMP‐9 in the HFHS and HFLS group were markedly higher than those detected in the ND group, which is consistent with the findings reported by Huang *et al*. 35.

Studies demonstrated that visfatin is related to CRC and breast cancer 38, 39. Moreover, the role of visfatin in the development of CRC has been attributed to one of several possible mechanisms. Visfatin contributes to the survival of tumor cells, which may result in cancer. SDF‐1 interacts with its only receptor CXCR4 to form a coupling molecular pair, which is closely related to signal transduction and migration of tumor cells. In addition, NF‐κB is also associated with cell proliferation, apoptosis and metastasis 26. Flores *et al.* 36 reported that TNF‐α promotes tumor cell growth and tumor infiltration via a CRC model. Using a colon cancer model, Liu *et al.* 37 demonstrated that TNF‐α and its downstream signaling pathways exert a pivotal effect on obesity‐induced CRC. In our study, the level of visfatin was significantly increased in the HFHS and HFLS groups. In addition, cell proliferation, migration and invasion were detected in colon cancer HCT116 cells, and there was obvious cell proliferation, greater numbers of HCT116 cell colonies and cell migration in the HFHS‐CM and HFLS‐CM groups versus the ND‐CM and SFM groups. Similarly, our results in colon cancer HCT116 cells were also observed by Kollmar *et al.* 38 and Yoshitake *et al.* 39.

In this study, we focused on high‐fat diet‐induced adipokines and cytokines, which promoted the progression (proliferation, colony formation, migration and invasion) of CRC HCT116 cells *in vitro*. However, we did not further verify which specific adipokines or cytokines are involved in this process. In addition, it is possible that elevated levels of free fatty acids in the serum may have stimulated the parameters investigated in the *in vitro* experiments. Furthermore, the molecular mechanism through which these adipokines or cytokines promote the proliferation of CRC warrants further investigation.

In summary, we found that high‐fat diet increases the number of adipokines and cytokines *in vivo*. Furthermore, we found that serum derived from mice fed an HFHS or HFLS that contains excess adipokines and cytokines promoted the progression (proliferation, colony formation, migration and invasion) of colon cancer HCT116 cells. Thus, high‐fat diet‐induced adipokines and cytokines may exert a positive effect on the development of CRC *in vivo* and *in vitro*.

## Conflict of interest

The authors declare no conflict of interest.

## Author contributions

JZ and SG conducted experiments and were responsible for data acquisition and manuscript writing. JL, WB, PZ and YH were responsible for data interpretation and data analysis. PL and YW helped with statistical analysis. QZ conceived and designed the study, and critically revised the manuscript for important intellectual content. All authors read and approved the final manuscript.
